# The Contribution of Childhood Adversity and Potentially Traumatic Events During Military Service to PTSD and Complex PTSD Symptoms Among Israeli Women Veterans

**DOI:** 10.1192/j.eurpsy.2023.430

**Published:** 2023-07-19

**Authors:** G. Zerach

**Affiliations:** Psychology, Ariel University, Ariel, Israel

## Abstract

**Introduction:**

Adverse childhood experiences (ACEs) and exposure to potentially traumatic events (PTEs) during military service are associated with mental health problems. However, knowledge about relative contributions of these factors to non-U.S women combat veterans’ posttraumatic sequalae is sparse.

**Objectives:**

To examine associations between ACEs, combat exposure, military sexual trauma (MST), potentially morally injurious events (PMIEs), posttraumatic stress disorder (PTSD) and complex PTSD symptoms among women veterans.

**Methods:**

A volunteer sample of Israeli women combat veterans (*n*=885) and non-combat veterans (*n*=728) responded to self-report questionnaires in a cross-sectional design study.

**Results:**

Combat veterans reported more total average ACEs, were more likely to experience 3 or more ACEs and specific ACEs of physical abuse and emotional neglect, as compared to non-combat veterans. Combat veterans also reported higher levels of combat exposure, PMIEs, higher prevalence of MST and higher levels of PTSD symptoms, but not CPTSD symptoms, as compared to non-combat veterans. Importantly, ACEs, combat exposure, MST-assault and PMIEs of betrayal predicted PTSD symptoms, while only ACEs and PMIEs of betrayal predicted complex PTSD symptoms.

**Conclusions:**

This study emphasized the relatively high exposure to PTEs and PTSD symptoms of women combat veterans as compared to non-combat veterans. Our findings also confirm prior studies demonstrating associations between ACEs, combat exposure, MST and mental health problems. Importantly, we demonstrated the unique contribution of betrayal based PMIEs and the differential associations of PTEs with PTSD and Complex PTSD symptoms among combat veterans.

**Disclosure of Interest:**

None Declared

